# TassDB2 - A comprehensive database of subtle alternative splicing events

**DOI:** 10.1186/1471-2105-11-216

**Published:** 2010-04-29

**Authors:** Rileen Sinha, Thorsten Lenser, Niels Jahn, Ulrike Gausmann, Swetlana Friedel, Karol Szafranski, Klaus Huse, Philip Rosenstiel, Jochen Hampe, Stefan Schuster, Michael Hiller, Rolf Backofen, Matthias Platzer

**Affiliations:** 1 Bioinformatics group, Albert-Ludwigs-University Freiburg, Georges-Koehler-Allee 106, 79110 Freiburg, Germany; 2 Genome Analysis, Leibniz Institute for Age Research - Fritz Lipmann Institute, Beutenbergstr. 11, 07745 Jena, Germany; 3 Bio Systems Analysis Group, Friedrich Schiller University Jena, Ernst-Abbe-Platz 1-4, D-07743 Jena, Germany; 4 Leibniz Institute for Natural Product Research and Infection Biology, Hans-Knöll-Institute, Systems Biology/Bioinformatics, Beutenbergstrasse. 11a, 07745 Jena, Germany; 5 Institute of Clinical Molecular Biology, Christian-Albrechts-University Kiel, Schittenhelmstrasse, 12, 24105 Kiel, Germany; 6 Department of General Internal Medicine, University Hospital Schleswig-Holstein, Campus Kiel, Schittenhelmstrasse, 12, 24105 Kiel, Germany; 7 Department of Bioinformatics, Friedrich Schiller University Jena, Ernst-Abbe-Platz 2, 07743 Jena, Germany; 8 Department of Developmental Biology, Stanford University, Stanford, CA 94305, USA; 9 Freiburg Initiative for Systems Biology (FRISYS), University of Freiburg, Schaenzlestrasse 1, 79104 Freiburg, Germany; 10 Centre for Biological Signalling Studies (bioss), University of Freiburg, Albertstr. 19, 79104 Freiburg, Germany

## Abstract

**Background:**

Subtle alternative splicing events involving tandem splice sites separated by a short (2-12 nucleotides) distance are frequent and evolutionarily widespread in eukaryotes, and a major contributor to the complexity of transcriptomes and proteomes. However, these events have been either omitted altogether in databases on alternative splicing, or only the cases of experimentally confirmed alternative splicing have been reported. Thus, a database which covers all confirmed cases of subtle alternative splicing as well as the numerous putative tandem splice sites (which might be confirmed once more transcript data becomes available), and allows to search for tandem splice sites with specific features and download the results, is a valuable resource for targeted experimental studies and large-scale bioinformatics analyses of tandem splice sites. Towards this goal we recently set up TassDB (Tandem Splice Site DataBase, version 1), which stores data about alternative splicing events at tandem splice sites separated by 3 nt in eight species.

**Description:**

We have substantially revised and extended TassDB. The currently available version 2 contains extensive information about tandem splice sites separated by 2-12 nt for the human and mouse transcriptomes including data on the conservation of the tandem motifs in five vertebrates. TassDB2 offers a user-friendly interface to search for specific genes or for genes containing tandem splice sites with specific features as well as the possibility to download result datasets. For example, users can search for cases of alternative splicing where the proportion of EST/mRNA evidence supporting the minor isoform exceeds a specific threshold, or where the difference in splice site scores is specified by the user. The predicted impact of each event on the protein is also reported, along with information about being a putative target for the nonsense-mediated decay (NMD) pathway. Links are provided to the UCSC genome browser and other external resources.

**Conclusion:**

TassDB2, available via http://www.tassdb.info, provides comprehensive resources for researchers interested in both targeted experimental studies and large-scale bioinformatics analyses of short distance tandem splice sites.

## Background

Alternative splicing (AS), a process which enables the production of multiple mRNA transcripts by the same gene via the variable inclusion of parts of the primary transcript, is very widespread in eukaryotes - almost all multi-exonic human genes are believed to undergo AS [1,2]. Thus, AS is a major contributor to the complexity and diversity of eukaryotic transcriptomes and proteomes. The splice variants produced can either exhibit different properties (e.g. half-life, translational efficiency), be translated into different protein isoforms with potentially different functions, or can be degraded via pathways such as the nonsense-mediated decay (NMD) [3]. AS can often be specific to a tissue type or developmental stage, and the majority of human AS events are believed to be regulated in this sense [1]. The regulation of AS has been shown to play an important role in several developmental processes in various organisms, and defects in AS can lead to diseases [4].

Subtle AS, involving splice sites separated by a distance of 2-12 nt, is an important, evolutionarily widespread subclass of AS [5]. Such AS is called subtle because the resulting mRNA isoforms differ by only a few nucleotides. While alternative acceptors (AA) and alternative donors (AD) together constitute about a third of all AS events in humans, subtle AS events comprise about a third of AA and AD events - for example, subtle events constitute 1,586 (38%) out of 4,179 AA events and 774 (28%) out of 2,728 AD events in the "alt events" track of the UCSC genome browser [6] for a combined total of 34% (2,360/6,907). Another reason for treating these events separately is that the mechanisms behind such events are likely different from those involving splice sites separated by larger distances - for example, the emergence of a second polypyrimidine tract can be observed for alternative acceptors separated by 8 or more nucleotides, and events which result in a frame-preserving difference of transcript length are seen to be more common than frame-shifting ones, once we move beyond a difference of 12 nt [7,8]. In the following, we shall use the notation *Δx *to denote a subtle splice event involving sites separated by *x *nucleotides, so for example, the class *Δ3 *shall be used to mean all GYNGYN and NAGNAG AS events (Y stands for C or T; N for A, C, G, or T), and so on.

It is a matter of debate as to what fraction of subtle AS events are truly functional, as opposed to being a result of a noisy process in which the spliceosome stochastically selects between nearby competing alternatives [5,9-11]. Consistent with estimations that a fraction of those subtle AS events is under purifying selection [12], there are several known cases where they result in functionally different protein isoforms or affect the translational efficiency when located in the untranslated regions (UTR) [5]. Moreover, subtle AS can also have a decidedly unsubtle effect in cases where a premature stop codon can be created, which is especially likely in cases where the splice sites are separated by a distance which is not a multiple of 3. Mutations that create frame-preserving tandem splice sites affecting the coding region are selected against [13], and in the case of *ABCA4 *(*Δ3 *acceptor, [14]) and *WT1 (Δ9 *donor, [15]) are associated with human disease. In the human *EDA *gene, AS at a conserved *Δ6 *donor leads to isoforms with distinct receptor binding specificity [16]. For more examples and further details regarding the functional consequences of subtle AS, the reader is referred to [5].

TassDB1 (TAndem Splice Site DataBase, version 1), the first database devoted to subtle AS, provides large collections of *Δ3 *donors and acceptors in eight species [17]. We have extended TassDB1 considerably, to create TassDB2, which provides a comprehensive collection of all human and mouse donors and acceptors in the *Δ2*-*Δ12 *range. We note that while TassDB provided data on 8 species, TassDB2 only includes 2 species, human and mouse. This is because the transcriptome coverage by ESTs/mRNAs in the remaining species was insufficient for detection of a non-negligible number of AS events involving the larger distances in the *Δ2*-*Δ12 *range. TassDB2 includes data on the conservation of the tandem motifs in five vertebrates (human, mouse, dog, chicken and zebrafish). Thus, TassDB2 provides comprehensive information on 22 event types, compared to 2 (NAGNAG and GYNGYN) in TassDB1. Thus TassDB2 is effectively a new database rather than just a simple extension. A user-friendly search interface features both a "quick search" mode, in which a user can search using gene symbol, as well as an "advanced search" mode, in which several different criteria can be specified by the user, and the possibility to download result datasets.

## Construction and content

### Data

TassDB2 uses an annotation pipeline based on transcript-to-genome mappings taken from the UCSC genome browser [18]. We used the RefSeq annotation as well as the UCSC 'knownGene' set for human (build hg18) and mouse (build mm9). The exon-intron structure as well as the protein-coding sequence (CDS) annotation was as per the UCSC annotation. Alternative tandem splicing events were identified using BLAST against all ESTs and mRNAs from the respective species as described in [19,20].

For each tandem splice site and the confirmed or putative AS event, TassDB2 contains the following data: the splice site motif, its genomic locus, its location in the transcript (5'/3'-UTR or CDS with intron phase 0/1/2), the (predicted) impact of the splice event on the protein, the sequences and length of the up-/downstream exon and the intron, and information about the ESTs/mRNAs that indicate usage (if any) of the splice sites. Their nomenclature is E/e for intron-proximal acceptor/donor (distal part of the tandem becomes exonic) and I/i for intron-distal ones (entire tandem becomes intronic). As the strength of the splice sites in a tandem often helps to distinguish between alternatively and non-alternatively spliced tandem motifs [7,9,20], we also computed splice site scores for both splice sites in each tandem [21].

TassDB2 holds splice site specific data as well as transcript-dependent data. Some features, such as the tandem motif (the two NAGs or GTNs, and the intervening sequence, if any - *Δ2 *being a special case, with motifs NAGAG and GTGTN), the genomic locus and the splice site scores, are independent of transcript annotation. However, other features such as intron phase, protein impact, EST confirmation and predicted targeting by NMD depend on the CDS annotation and the exon-intron structure of the transcript. The features "protein impact", "position in protein", and "NMD e/i" are all computed separately for each transcript, as detailed in the original TassDB paper [17]. Targeting by NMD is predicted in the usual manner - for each transcript with a stop position upstream/5' of the last exon-exon junction, we calculated the nucleotide distance between the stop position (corresponding to the given splice variant) and the position of the last exon-exon junction, and if this distance was greater than 50, targeting by the NMD pathway was predicted.

### Database Design

**Figure 1:**
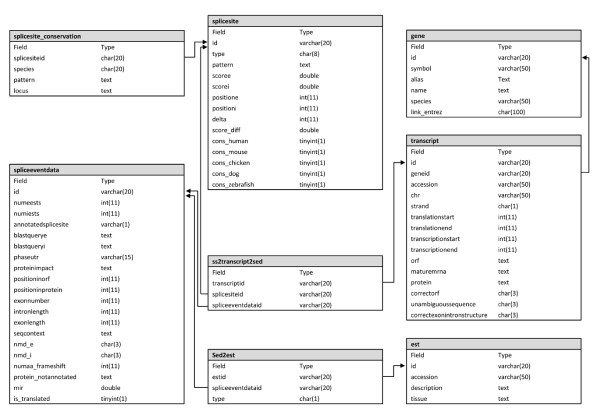
**The database scheme of TassDB2**. The figure shows the details of all eight tables, and their interdependencies.

The web-frontend to TassDB2 is created in HTML with PHP and JavaScript. The data is stored in a relational database, running under the MySQL database system. The data is primarily organized in the database tables *splicesite, spliceeventdata*, and *transcript *(Figure 1).

The table *splicesite *contains sequence-dependent information such as the genomic locus, the splice site pattern with its sequence context, the splice site scores, and conserved tandem sequences (if available) in human/mouse, chicken, dog, and zebrafish. All transcript-dependent data is stored in table *spliceeventdata*: the transcripts which have the tandem site in their exon-intron structure, the annotated splice site, the number of ESTs for each (potential) tandem splice variant along with the two BLAST queries used to find the ESTs, the predicted protein impact, and the NMD prediction. The table *transcript *contains the information on the transcripts that is independent from the splice sites. The three main tables are linked through the *ss2transcript2sed *table.

Additionally, each splice site is linked to information on its gene (table *gene*), and its conservation in other species (table *splicesite_conservation*; species are human, mouse, dog, chicken, zebrafish, representing the major vertebrate clades). Here, conservation simply means that both species contain tandem splice sites - the neighbouring nucleotides need not be conserved. The splicing events are linked to their supporting ESTs in the table *est*. The user interface contains links giving a detailed description of each data field.

Summary statistics of human tandem splice sites in TassDB2 are given in Table 1.

**Table 1:** Statistics of human tandem splice sites in TassDB2.

	Donors #*%*				Acceptors #*%*			
		
Delta	Tandem splice sites		Confirmed* alternative		Tandem splice sites		Confirmed alternative	
	
2	9,825	*1.9*	164	*1.7*	11,135	*6.7*	252	*2.3*
3	11,164	*2.2*	166	*1.5*	12,542	*7.5*	2,272	*18.1*
4	130,104	25.3	955	*0.7*	11,852	*7.1*	961	*8.1*
5	36,643	*7.1*	269	*0.7*	11,314	*6.8*	609	*5.4*
6	54,142	*10.5*	275	*0.5*	16,495	*9.9*	396	*2.4*
7	45,161	*8,8*	150	*0.3*	17,290	*10.4*	179	*1.0*
8	42,670	*8.3*	150	*0.4*	15,386	*9.2*	175	*1.1*
9	46,688	*9.1*	249	*0.5*	18,645	*11.2*	212	*1.1*
10	47,092	*9.1*	217	*0.5*	17,294	*10.4*	160	*0.9*
11	44,831	*8.7*	157	*0.4*	15,832	*9.5*	123	*0.8*
12	46,654	*9.1*	267	*0.6*	18,819	*11.3*	204	*1.1*
Total	514,974	*100.0*	3,019	*0.6*	166,604	*100.0*	5,543	*3.3*

* Tandem splice sites are considered confirmed if both splice forms have at least one supporting EST/mRNA.

## Utility and Discussion

While several databases on AS ignore subtle AS events, others (e.g. [6,22-25]) contain them but provide no straightforward way of searching for them via the user interface. To the best of our knowledge, none of the existing databases on AS provide the option of searching for alternative donors or acceptors separated by a specific distance. While some of the databases contain a comparable number of subtle AS events (Additional file 1), these have to be teased out from the raw data using computer programs, whereas TassDB2 lets the user search for them in a straightforward manner. Moreover, it is the only database to contain an exhaustive list of putative tandem splice sites, as these surely contain a subset which shall be revealed as being alternative, once more transcript data (such as RNA-seq data from next-generation sequencing platforms) is available. We demonstrated this for NAGNAG events in our work on NAGNAG AS prediction [26], where we experimentally confirmed the existence of subtle AS for many putative events (chosen on the basis of our predictions).

### User interface - quick search and advanced search

We anticipate that the most frequent use of TassDB2 will be a search for tandem splice sites of a given gene. Therefore, TassDB2 provides a^"^quick search" interface where a user need only specify a gene symbol or a transcript accession number, and the entire information of both confirmed and unconfirmed tandem splicing events for this gene is displayed.

**Figure 2:**
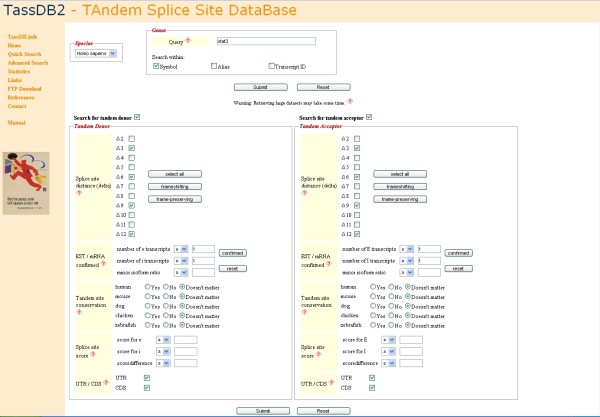
**The advanced search interface of TassDB2**.

Often, however, users might be interested in information which requires a selection of tandem splice sites with specific features. To address this, TassDB2 also provides an "advanced search interface" (Figure 2) where the search can be restricted using one or more of the following features: (i) *Δ *- the distance between the splice sites, (ii) frame-preserving or/and frame-shifting, (iii) number of ESTs/mRNAs that match both splice forms, (iv) "minor isoform ratio", that is the fraction of ESTs/mRNA that support the minor isoform, (v) tandem site conservation in any or all of five organisms (human, mouse, dog, chicken, and zebrafish) (vi) splice site scores for the two splice sites, (vii) the difference in the splice site scores, and (ix) location in the UTR or CDS. Thus, it is easy to formulate queries such as: "Show all confirmed *Δ3 *events with a minor isoform ratio ≥ 0.4", "Show all tandem splice sites where both splice forms are represented by at least two ESTs/mRNAs and the minor isoform ratio is ≥ 0.15" or "Show all confirmed frame-shifting tandem donors which are located in the CDS". Additionally, the search can be restricted to certain genes.

### User interface - reporting results

The result of the search consists of two parts: (i) a summary table listing the affected genes and their number of tandem splice sites of each type, and (ii) detailed tables containing information regarding the individual tandem splice sites. These detailed result tables also provide links to the ESTs/mRNAs for both splice forms as well as links to the UCSC genome browser. If the transcript specific data differ between transcripts, TassDB2 shows detailed result tables with more than two columns. Features that differ between transcripts are shown in black while those that are identical in all transcripts are shown in grey color.

### Examples

**Figure 3:**
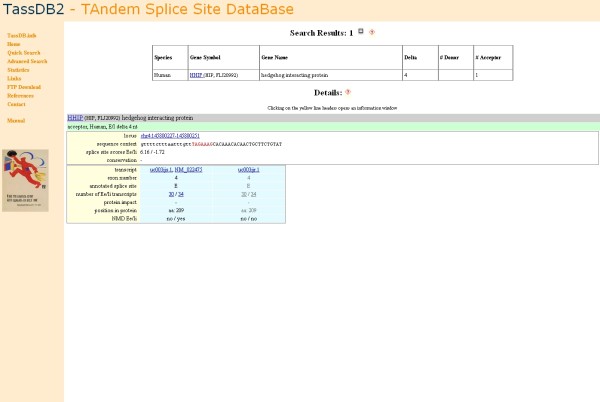
**Result page for all confirmed tandem splice sites in the human *HHIP *gene**.

Searching for all confirmed tandem splice sites in the gene *HHIP *(hedgehog interacting protein) in human leads to the result page shown in Figure 3: *HHIP *has one confirmed *Δ4 *tandem acceptor event, with the upstream and downstream acceptor supported by 30 and 34 ESTs/mRNAs, respectively. The event is predicted to lead to targeting by NMD according to one of the representative transcripts (uc003ijs.1, NM_022475), but not according to the other (uc003ijr.1).

While AS has now been established as a widespread phenomenon and a substantial contributor to the complexity of eukaryotic transcriptomes and proteomes, it is still a matter of great debate as to how many AS events are truly functional [9-12,27,28]. The literature regarding this question is the motivation behind providing the options for searching by splice site score difference and minor isoform ratio in TassDB2. It has been observed that comparable splice site strength is often indicative of both splice sites in a tandem being used, whereas a higher fraction of ESTs/mRNA supporting the minor isoform is a good test of whether the event is likely to be genuine AS events rather than just noise [29,30]. As an example, searching for all confirmed tandem splicing events with a minor isoform ratio of ≥ 0.45 yields 300 results, and increasing the threshold of supporting ESTs/mRNAs to ≥ 10 for each variant yields 170 results.

The TassDB2 resource also includes the BayNAGNAG webserver (available at http://www.tassdb.info/baynagnag/), which uses Bayesian networks to predict the splicing outcome at NAGNAG tandem splice sites in an EST/mRNA independent way based on splice site features [26].

## Conclusions

TassDB2 is a comprehensive resource for information regarding subtle AS. Users can easily search for individual genes, as well as by various criteria corresponding to different features of the tandem splice sites. Some of the criteria can be used to enrich for splicing events which are likely to have functional significance. The results can be downloaded for further exploration, and flat files have also been made available for those who wish to carry out their own large-scale bioinformatics studies (see Additional files 2 and 3 for all confirmed subtle AS events in Human and Mouse, respectively). Thus TassDB2 should be a very useful resource for scientists interested in subtle AS.

## Availability and requirements

TassDB2 is freely available for online use at http://www.tassdb.info

TassDB2 can be used via any standard internet browser.

## Authors' contributions

RS participated in database design and testing, found the alternative tandem splice sites, and drafted the manuscript. TL participated in database design and testing, and implemented several parts of the front-end and back-end of TassDB2. NJ improved the implementation of the database, and optimized it for faster searches. UG participated in database design and performed extensive testing. MH, SF and KS participated in database design and testing, and provided data and scripts. SF and UG took part in front-end improvement. KH participated in database design and testing. PR, JH, SS, MH, RB and MP designed and supervised the project, participated in database design and testing, and manuscript preparation. All authors read and approved the final manuscript.

## Supplementary Material

Additional file 1 **The abundance of subtle AS events in some AS databases**. Plots comparing the number of human subtle AS events in TassDB2, ASTD, and the alternative splicing track of the UCSC genome browser. Click here for file

Additional file 2 **Confirmed subtle AS events in Human**. All subtle AS events in Human with at least 1 EST/mRNA per splice variant. Click here for file

Additional file 3 **Confirmed subtle AS events in Mouse**. All subtle AS events in Mouse with at least 1 EST/mRNA per splice variant. Click here for file
