# Supplementary material for: TassDB2 - A comprehensive database of subtle alternative splicing events
Source: BMC Bioinformatics. 2010 Apr 29;11:216. doi: 10.1186/1471-2105-11-216 (PMC2878309; doi:10.1186/1471-2105-11-216)
Supplement: Additional file 1 — The abundance of subtle AS events in some AS databases. Plots comparing the number of human subtle AS events in TassDB2, ASTD, and the alternative splicing track of the UCSC genome browser. [file 1471-2105-11-216-S1.DOC]

(a)


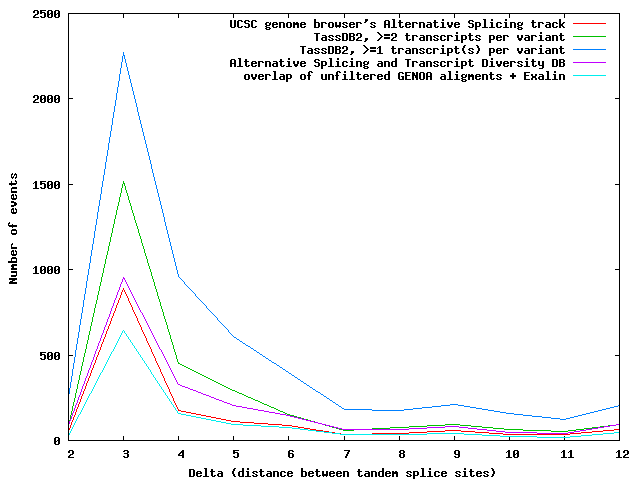


(b)


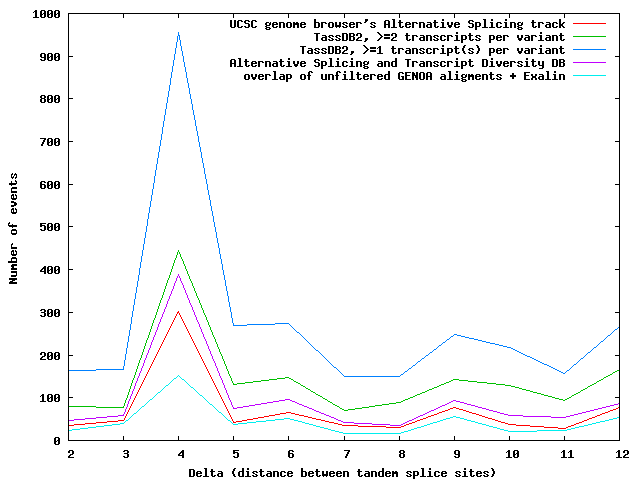


Subtle alternative splicing events in some publically available databases. The number of subtle alternative (a) acceptors and (b) donors in Human according to the alternative splicing track of the UCSC browser, TassDB2, the Alternative Splicing and Transcript Diversity database, and inferred from the overlap of unfiltered Genoa alignments with Exalin (personal communication by Ralf Bortfeldt, ref. Bortfeldt et. al, BMC Genomics 2008, PMID 18447903). Events from TassDB2 have been shown for two different filtering criteria – with ≥1 supporting transcripts per variant (“confirmed” events), or ≥2 supporting transcripts per variant (showing good agreement with the numbers both other databases). The counts for the other two databases were obtained using Perl scripts to parse the publically available flatfiles.
